# Bio-Based Polyhydroxyalkanoate (PHA) Blends for 3D Printing: Rheological, Mechanical, Biocompatibility, and Biodegradation Properties

**DOI:** 10.3390/polym17111477

**Published:** 2025-05-26

**Authors:** Michal Ďurfina, Nafiseh Babaei, Zuzana Vanovčanová, Jozef Feranc, Vojtech Horváth, Ida Vašková, Ján Kruželák, Katarína Tomanová, Roderik Plavec

**Affiliations:** 1 Institute of Natural and Synthetic Polymers, Faculty of Chemical and Food Technology, Slovak University of Technology in Bratislava, Radlinského 9, 812 37 Bratislava, Slovakia; michal.durfina@stuba.sk (M.Ď.); zuzana.vanovcanova@stuba.sk (Z.V.); jozef.feranc@stuba.sk (J.F.); vojtech.horvath@stuba.sk (V.H.); ida.vaskova@stuba.sk (I.V.); jan.kruzelak@stuba.sk (J.K.); katarina.tomanova@stuba.sk (K.T.); roderik.plavec@stuba.sk (R.P.); 2 Polymer Institute of the Slovak Academy of Sciences, Dúbravská cesta 9, 845 41 Bratislava, Slovakia

**Keywords:** PHA, 3D printing, bio-based, home compostable, flexible, tissue engineering

## Abstract

This study develops highly flexible, biodegradable polymer blends using bio-based polyhydroxyalkanoate (PHA) polymers for Fused Deposition Modeling (FDM) 3D printing. A Design of Experiment (DoE) approach optimized blend compositions by varying crystallinity levels of three PHAs, processed via twin-screw extrusion. Rheological analysis revealed that PHA blends exhibited 30–50% lower viscosity than PLA at low shear rates, ensuring improved processability. Tensile testing confirmed favorable mechanical properties, with elongation at break exceeding 2000%, significantly surpassing PLA (29%). Differential scanning calorimetry (DSC) indicated partial miscibility and crystallinity reductions of up to 50%, influencing printability. Optimized 3D printing parameters demonstrated minimal warping for blends with crystallinity below 18%, ensuring high-dimensional stability. During home composting tests, PHA blends showed significant degradation within two months, whereas PLA remained intact. Scanning electron microscopy (SEM) confirmed microbial degradation. Cytotoxicity tests demonstrated that the blends were non-toxic, supporting applications in tissue engineering. These findings highlight the potential of PHA-based blends as sustainable, high-performance materials for biomedical, packaging, and environmental applications.

## 1. Introduction

Three-dimensional (3D) printing, also known as additive manufacturing (AM), is an advanced fabrication technology that enables the production of intricate structures with complex architectures. This method has revolutionized production by enabling rapid engineering design, cost efficiency, and improved product quality control [1,2]. Moreover, 3D printing has transformed the manufacturing sector, shifting from prototype development to the rapid production of final products [3,4]. Unlike conventional manufacturing, which often involves subtractive techniques that generate excess waste, 3D printing follows an additive approach, constructing objects layer by layer with precise material usage. This minimizes material waste, enhances sustainability and production efficiency, and makes 3D printing a highly advantageous and environmentally responsible solution [5,6].

Fused Deposition Modeling (FDM) is among the most widely utilized additive manufacturing techniques. In this method, a thermoplastic filament is heated and extruded through a nozzle, building objects layer by layer [7,8]. Compared to other 3D printing techniques, FDM offers multiple advantages, including a wide selection of material options, ease of operation, and relatively low production costs. These characteristics make FDM particularly suitable for industrial applications and small-scale manufacturing [9,10].

FDM printing commonly utilizes thermoplastics such as polylactic acid (PLA), acrylonitrile butadiene styrene (ABS), and thermoplastic polyurethanes (TPU) [11,12,13]. Bio-based polymers like PLA have gained attention as sustainable alternatives to petroleum-derived plastics. PLA is favored for its printability, high mechanical performance, and biodegradability [13,14]. However, PLA exhibits limited biodegradability under natural environmental conditions and requires industrial composting for efficient decomposition [15,16,17]. Studies indicate that PLA undergoes minimal degradation after 4–5 months of soil burial [18,19,20].

ABS and TPU are also widely used in FDM due to their excellent mechanical properties. TPU is known for its high flexibility and abrasion resistance, while ABS offers strength and heat resistance. Both materials are easily extruded and compatible with standard FDM printers, making them popular choices for diverse applications [21,22]. However, as petroleum-based, non-biodegradable polymers, they pose challenges in waste management and long-term sustainability [23,24].

These environmental concerns highlight the need for materials that combine excellent 3D printing capabilities with enhanced sustainability. Developing biodegradable alternatives could mitigate the impact of plastic waste accumulation in landfills and ecosystems, addressing long-term pollution and waste management challenges [25,26].

Polyhydroxyalkanoates (PHAs) have attracted significant attention due to their sustainability, biodegradability, and potential applications in fields such as biomedical engineering, agriculture, food packaging, and cosmetics. PHAs are biopolymers produced by bacterial fermentation and degrade under aerobic and anaerobic conditions [27,28]. Among PHAs, polyhydroxybutyrate (PHB) is one of the most widely produced and commercially available variants. PHB exhibits properties comparable to polypropylene, including crystallinity, melting and glass transition temperatures, strength, and modulus. It can be processed using conventional melt processing techniques, such as 3D printing, extrusion, injection molding, and thermoforming [29,30]. Additionally, PHB demonstrates good moisture resistance and superior oxygen barrier properties compared to polyolefins, making it a promising alternative to synthetic polymers [31]. However, native PHB has limitations such as brittleness and a narrow processing window [32].

To address this issue, PHB can be blended with more flexible polymers like polyhydroxy butyrate-co-valerate (PHBV), polybutylene succinate-co-adipate (PBSA), or polycaprolactone (PCL) or modified with toughening agents to improve flexibility and processability [33,34,35]. Hlavacikova et al. investigated biodegradable materials suitable for FDM 3D printing by blending PHB and PHBV with PLA and modifying additives using a twin-screw extruder. The resulting blends exhibited improved elongation at break and lower crystallization temperatures compared to pure PHB or PHBV. While unmodified PHB and PHBV filaments showed poor flow behavior and warping, the prepared blends demonstrated enhanced flowability and enabled high-quality 3D printing [36].

Furthermore, PHBVs with medium hydroxy valerate (HV) content have gained interest due to their balanced flexibility and mechanical stability, addressing brittleness issues associated with high HB content [37,38,39]. The presence of medium HV concentrations improves 3D printing performance while maintaining eco-friendly properties [40,41]. For example, Yichao Ma et al. developed biodegradable active packaging materials by blending PLA with PHB. The addition of PHB enhanced the mechanical properties, particularly elongation at break and toughness, thereby overcoming PLA’s inherent brittleness. Their study demonstrated that the PLA-PHB blend effectively preserved salmon freshness, showcasing its potential as an eco-friendly packaging alternative [42].

While biodegradable polymers have gained significant attention, the influence of blending PHAs with different crystallinity levels on 3D printing performance remains largely unexplored. This study aims to fill this gap by developing and characterizing fully bio-based and biodegradable polymer blends with tailored crystallinity to improve printability, mechanical strength, and environmental sustainability.

Herein, PHA polymers were blended using a twin-screw extruder, and the Design of Experiment (DoE) approach was applied to systematically assess how composition influences material properties. Crystallinity and glass transition temperatures were analyzed through differential scanning calorimetry (DSC), while mechanical properties and 3D printing performance were evaluated in detail. This study further examines the role of medium- and low-crystallinity PHAs in shaping rheological behavior, printability, and biodegradability under real home composting conditions. Additionally, biocompatibility assessments, including agar diffusion and contact cytotoxicity tests, were conducted to explore the suitability of these flexible, 3D-printable materials for applications in biomedical engineering, sustainable packaging, and agriculture.

## 2. Materials and Methods

### 2.1. Materials

Three types of Polyhydroxyalkanoates (PHAs) were delivered by the company Panara a.s., Nitra, Slovakia: PHA1—polyhydroxyalkanoate P3HB with high crystallinity (52%), Tg = 5.1 °C, Tm = 166.7 °C, and Mw 219,000 g/mol, in powder form. PHA2—amorphous polyhydroxyalkanoate P3HB4HB with 0% crystallinity, Tg = -14.9 °C, and Mw 250,000 g/mol, Tm not detectable on DSC. PHA3—polyhydroxyalkanoate P3HB3HH with medium crystallinity (36.2%), Tg = 2.4 °C, Tm = 148.6 °C, and Mw 230,000 g/mol. The PLA filament for 3D printing was delivered by the company Turtle, s.r.o., Nitra, Slovakia, and was considered as the reference material for 3D printing.

The cells used were gingival fibroblasts (GFs). This study was conducted in accordance with the Declaration of Helsinki and approved by the Ethics Committee of University Hospital Bratislava—Academician Ladislav Dérer Hospital (protocol code 06/2022, date of approval 19 January 2022). GFs were isolated and stored in liquid nitrogen at the Institute of Medical Biology, Genetics, and Clinical Genetics at Comenius University (Bratislava, Slovakia). In vitro cultivation of GFs was performed in a humidified 6% CO_2_ atmosphere at 37 °C using Dulbecco’s modified Eagle’s medium (DMEM) supplemented with 10% fetal bovine serum (FBS), and antibiotics (Penicillin 100 U/mL, Streptomycin 100 µg/mL, Amphotericin B 0.25 µg/mL). The cells were passaged at least once a week.

### 2.2. Methods

#### 2.2.1. Blend Preparation

(a) Design of Experiment

As the first step, the various formulations of blends containing three different types of PHAs were investigated to determine the optimal compositions for materials suitable for Fused Deposition Modeling (FDM) 3D printing. The primary objective was to develop materials that are 100% bio-based, home-compostable, biocompatible, and flexible. For this purpose, the Design of Experiment (DoE) methodology was employed. A two-factor, five-level DoE was designed, and the factors were chosen as follows:

(1) $$x_{1}=\frac{PHA2}{PHA1+PHA3}$$

(2) $$x_{2}=\frac{PHA3}{PHA1}$$

The first factor (*x*_1_*)* was the *PHA*2-to-(*PHA*1 + *PHA*3) mass ratio (*PHA*2/(*PHA*1 + *PHA*3)), and second factor of the experiment (*x*_2_) was the *PHA*3-to-*PHA*1 mass ratio. Table 1 presents the encoded values of both factors based on their actual real values, and Table 2 presents the composition in encoded values of all prepared blends. The limit values in coded levels −1.141 and 1.141 correspond with the limit composition of the samples, while the mean value is located at the coded coordinate 0. The confidence interval for the evaluation of results was 95%.

All measured and evaluated properties were analyzed using the DoE methodology. The regression coefficient of Equation (3) was calculated, and an Analysis of Variance (ANOVA) was performed for the DoE.

(3) $$Y=b0+b1.x1+b2.x2+b12.x1.x2+b12.x1.x2+b11.{x1}^{2}+b22.{x2}^{2}$$

Parameters *x*1 and *x*2 represent independent DoE factors, and regression coefficients *bi*, *bii*, and *bij* were calculated for each property from the statistical and regression analysis together with the calculation of critical values used for statistical significance evaluation.

In general, from an ANOVA analysis for all the tests in this paper, the important parameters will be discussed as follows:

F1–Fischer—Snedecor criteria for testing of significance of the linear part of Equation (1) for the given property;
F2–Fischer—Snedecor criteria for testing of significance of the non-linear part of equation (1) for the given property;
FLF–Fischer—Snedecor criteria for testing of significance of Equation (1) accuracy (lack of fit criteria);
sE+/−—Experimental standard deviation;
sLF+/−—Lack of fit standard deviation (inaccuracy of regression Equation (1)).

(b) Experimental Blending

All blending components were introduced into the hopper of a laboratory twin-screw extruder (Labtech, Thailand) with a screw diameter of 16 mm and a length-to-diameter (L/D) ratio of 40. The screw geometry incorporated three kneading zones, and atmospheric venting was positioned at the 38D mark on the barrel. Extrusion was conducted at a screw speed of 150 RPM, with the temperature profile along the barrel set to 70-120-170-180-190-190-190-180-175-170 °C from the hopper to the die.

The extruded melt was cooled in a water bath maintained at 20 °C. After exiting the water bath, surface moisture was removed by vacuum suction, and the strand was pelletized using a pelletization cutter. The pellets were then dried in a hot air oven at 50 °C for 24 h before further processing.

#### 2.2.2. Rheology Measurement

The samples were placed in the biconical chamber of the oscillatory rheometer, and the testing program was initiated. After sealing the chamber, the temperature was set to 180 °C, and the samples were preheated for 1.5 min at an oscillation angle of 6° and an oscillation frequency of 60 CPM. Following the preheating stage, the temperature was adjusted to 160 °C, and the flow curve measurement began at the same oscillation frequency of 60 CPM. The oscillation angle was gradually increased from 2° to 50°, resulting in shear rates ranging from 1.34 to 40 s^−1^. The complex viscosity was recorded using the rheometer software (Enterprise Online Manager software), and the relationships between complex viscosity and shear rate were evaluated for all samples.

Rheological analysis was additionally employed to assess the processing stability of the polymer blends. To this end, the complex viscosity of each sample was measured following a controlled thermo-mechanical loading procedure using an oscillatory rheometer. The specimens were introduced into a biconical test chamber, which was subsequently sealed, and the system temperature was adjusted to 200 °C. A static preheating phase of 0.5 min was applied to ensure complete melting of the samples. Thereafter, oscillatory deformation was initiated at a frequency of 50 cycles per minute (CPM) with an oscillation angle of 60°. Under these conditions, the evolution of complex viscosity was monitored over 10 min. The viscosity value recorded at the 4 min mark was a key indicator of processing stability. A lower complex viscosity at this stage was interpreted as indicative of reduced resistance to thermo-mechanical degradation and thus of lower processing stability.

#### 2.2.3. Thermal Properties Measurements

The thermal properties of all blends were determined using a differential scanning calorimeter (DSC 1, Mettler-Toledo Inc., Greifensee, Switzerland). Key thermal characteristics, including the glass transition temperature (Tg), crystallization temperature (Tc), and melting temperature (Tm), were evaluated from measurements conducted on blend pellets.

A sample weighing 10–15 mg was placed in a standard aluminum pan and inserted into the measurement chamber, while an empty aluminum pan was used as a reference. Nitrogen was employed as the inert gas with a flow rate of 50 mL/min. The measurement protocol was as follows:

- Isothermal hold at −70 °C for 3 min;
- First heating cycle: −70 °C to +200 °C at a heating rate of 10 K/min;
- Isothermal hold at +200°C for 3 min;
- Cooling cycle: +200 °C to −70 °C at a cooling rate of 10 K/min;
- Isothermal hold at −70 °C for 3 min;
- Second heating cycle: −70 °C to +200 °C at a heating rate of 10 K/min.

The heat flow dependency on temperature was recorded as the output of the measurement. All investigated thermal parameters were analyzed using DSC curves and processed with the SW STARe 16.40 evaluation software (Mettler-Toledo Inc.). Flow curves for all samples were recorded at 160 °C. Complex viscosity as well as the plastic and elastic parts of viscosity were evaluated for all 13 blends and the PLA standard.

#### 2.2.4. Preparation of Filaments for 3D Printing

Filaments suitable for Fused Deposition Modeling (FDM) 3D printing were produced from pre-dried pellets using a single-screw extruder (Plasticorder Brabender, Duisburg, Germany). The extruder was equipped with a 19 mm diameter screw, an L/D ratio of 25, and a compression ratio of 1:2. The extrusion process was conducted at a screw speed of 20 RPM, with a barrel temperature profile ranging from 160 °C to 190 °C (from the hopper to the die).

The extruder head was fitted with a round die of 2.0 mm diameter, and the extruded filaments were cooled in a water bath maintained at 45 °C. The filament diameter was regulated through a variable pulling speed on the pull-off device, ensuring a final filament diameter of 1.75 mm ± 0.15 mm.

#### 2.2.5. 3D Printing Procedure

All prepared samples in filament form were tested for 3D printability, and all specimens for mechanical testing were fabricated using 3D printing technology. For this purpose, an FDM 3D printer (Ender 3, Creality, Shenzhen, China) was employed. The printer was modified to enable printing with elastic filaments, featuring a direct-drive extruder and a dual 4010 cooling fan.

All samples were printed using a standard 0.4 mm nozzle on a textured PEI print bed, which was not heated during the printing process (resulting in a bed temperature range of 23–29 °C). Three-dimensional lac spray was applied to enhance bed adhesion when necessary.

The print parameters were as follows:

- Cooling fan speed: 100%;
- Print speed: 10 mm/s (0.7–0.5 mm^3^/s);
- Layer height: 0.2 mm.

**Figure 1:**
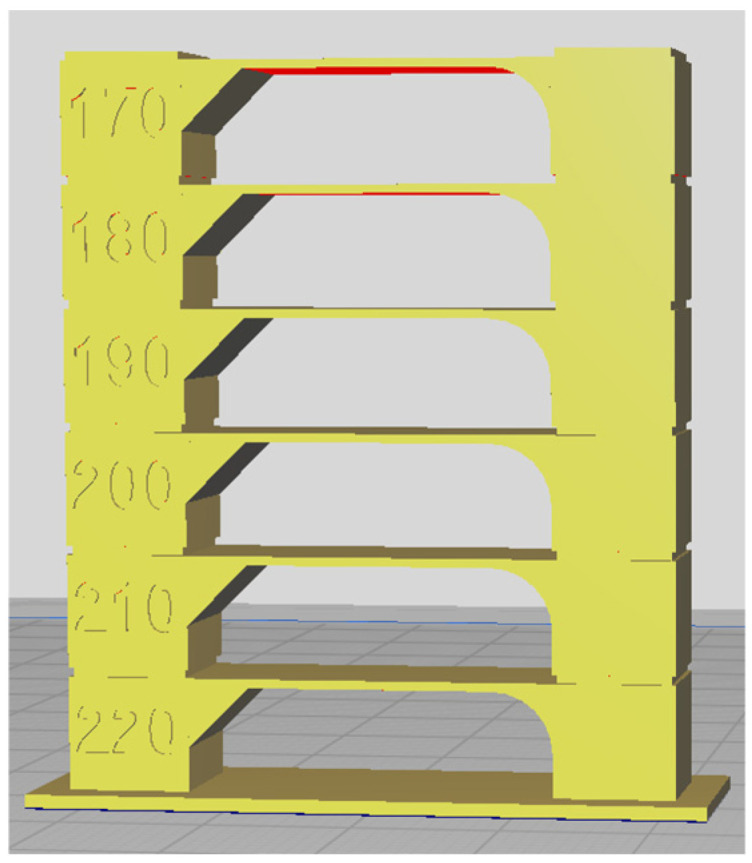
Temperature tower for optimal 3D printing temperature determination—slicer-designed model for 3D printing.

Each sample was 3D printed at optimal temperature, determined through temperature tower printing. Using FDM technology, temperature towers were printed for each biodegradable PHA polymer blend. For each material, a temperature tower was fabricated within a temperature range of 220 °C to 170 °C, with a temperature gradient of 10 °C per printed layer (Figure 1). The optimal printing temperature and overall print quality were assessed by analyzing geometric features such as interlayer bridges, overhangs, and the rounding of the outer wall.

The temperature tower consists of multiple levels, with each level printed at a different temperature. The first level was printed at 220 °C, and each subsequent level was printed at a temperature 10 °C lower until a printing temperature of 170 °C was reached. The quality of each level was visually inspected, and the optimal printing temperature for the polymer blend was determined based on the best-performing level. The design of the temperature tower is shown in Figure 1.

#### 2.2.6. Warping Test

**Figure 2:**
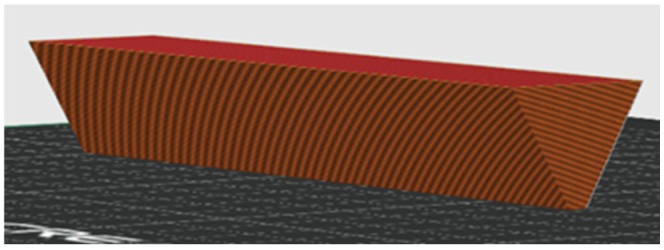
Triangular prism for warping test A—slicer model for 3D printing.

The warping tendency of each prepared sample was evaluated by 3D printing a specially designed test object (Figure 2) with a low contact surface at the optimal printing temperature. The printed object had a triangular cross-section prism shape, with an apex angle of 60° and a prism length of 6 cm.

The printing procedure was as follows:

- The first layer, with a width of 0.4 mm and a thickness of 0.2 mm, was applied to the bed, forming the apex of the triangular cross-section of the prism.
- Each subsequent layer was wider, ensuring that the apex angle remained at 60°.
- The height of each printed layer was 0.2 mm.

With each additional layer, the bending force acting on the bottom layer, due to the volumetric shrinkage of the cooled material, caused the edges of the prism to lift away from the build plate (bed). The printing process was monitored to determine at which layer the edges of the bottom layer detached from the bed. No warping effect was observed if the number of layers reached the maximum (43 layers) without detaching the edges of the bottom of the prism from the bed.

After each printing procedure, the build plate was thoroughly cleaned with water and degreased with isopropanol (IPA). The warping effect was evaluated based on the number of printed layers at which the first detachment from the bed was observed.

#### 2.2.7. Tensile Test

For the test, dog-bone tensile bars (ISO 37, Type 2) [43] were 3D printed using an FDM 3D printer. For each polymer blend, 8 tensile bars were printed at the optimal printing temperature with a layer height of 0.2 mm. Each sample was then tested using a universal testing machine (Zwick Roell, Ulm, Germany) according to ISO 37 [43]. The grip separation was set to 50 mm, with the contact extensometer working distance set to 30 mm. The cross-head speed was set to 50 mm/min. The yield stress, tensile strength at break, Young’s modulus, and tensile strength at break were evaluated from the tensile curves.

Tensile tests were also conducted on 3D filaments. The same universal testing machine was used for testing the filaments as for the tensile bars. The grip separation was set to 20 mm, with a cross-head speed of 50 mm/min. Elongation was determined from the grip separation at the point of break.

#### 2.2.8. Bending Test

For the determination of Young’s modulus in bending mode (flexural modulus), the three-point bending test was applied according to ISO 178 [44]. The specimens, in a bar shape, were printed using an FDM 3D printer at the optimal temperature, with dimensions of 80 × 10 × 4 mm. The three-point bending test was performed on a universal testing machine (Zwick Roell, Ulm, Germany) by ISO 178 [44]. The distance between the support points was set to 64 mm, and the load was applied using a load pin at the center of the span, with a cross-head speed of 5 mm/min. During the test, the stress–strain dependency of the specimen was recorded, and the flexural modulus was determined according to ISO 178 [44].

#### 2.2.9. Hardness Testing

The same specimens used for the bending test were employed for hardness measurements according to ISO 868 [45]. For each sample, 3 measurements were performed on each of the 8 specimens, resulting in a total of 24 measurements. The hardness was measured using the Shore D scale.

#### 2.2.10. Impact Strength Testing

The same 3D-printed bars used for the bending and hardness tests, with dimensions of 80 × 10 × 4 mm, were utilized for Charpy impact strength testing according to ISO 179-1 [46]. For each polymer blend, 5 bars were printed at the optimal printing temperature with a layer height of 0.2 mm. The test was performed using an impact pendulum tester (Instron CEAST 9050, Turin, Italy). The specimens were unnotched, and before testing, all specimens were cooled to −30 °C for 30 min. The potential energy of the pendulum used was 5 J.

#### 2.2.11. Home Compostability in Real Conditions

**Figure 3:**
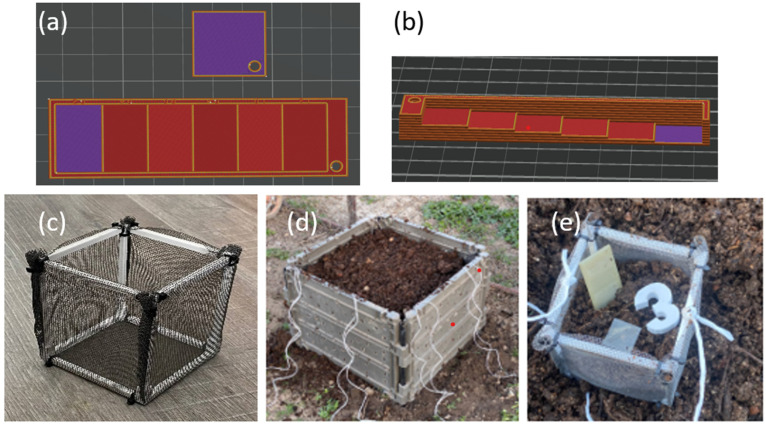
(**a**) Slicer models of 3D-printed samples in square and rectangular shape from (**a**) top view and (**b**) side view (to show increase in thickness) and (**c**–**e**) conditions for composting test according to experiment arrangement.

The compostability test was conducted under real conditions using a home compost system, specially designed with varying wall thicknesses (increasing from 0.2 mm to 1.2 mm), and a sample with a thickness of 0.2 mm in a square shape was 3D printed from selected polymer blends as well as from standard PLA for reference (the slicer models of the 3 printed specimens are shown in Figure 3a,b.

Each specimen was placed separately in a perforated cage with dimensions of 20 × 20 × 20 cm (Figure 3c), along with a specific marker for identification. The specimens were positioned in the center of the cage, surrounded by substrate from a home composter (Figure 3d). These cages were then evenly distributed in a regular composter (dimensions: 1 × 1 × 1 m), placed at the middle height, and surrounded by the same substrate (Figure 3e). The composter was left outdoors in the garden, uncovered.

Each week, fresh vegetable scraps and cut-offs were added to the composter and incorporated into the substrate. If the top layer became dry, the composter was thoroughly watered. The composting process took place over two months, with average temperatures ranging from 0 to 15 °C at night and 10 to 20 °C during the day.

At the end of the experiment, the specimens were carefully removed from the compost and cleaned under water. Decomposition was evaluated visually, and surface destruction was analyzed using scanning electron microscopy (SEM).

#### 2.2.12. Scanning Electron Microscopy (SEM)

Scanning electron microscopy was used for surface observation of samples before and after the home compostability test. Surface changes on the tested specimens before and after composting were observed using JEOL F 7500 SEM (JEOL, Tokyo, Japan). Samples were coated by a gold/platinum alloy using Balzers SCD 050 (Balzers AG, Balzers, Liechtenstein) sputtering equipment.

#### 2.2.13. Cytotoxicity Testing

The agar diffusion test was performed in accordance with ISO 10993-5 [47] to evaluate cytotoxicity. Gingival fibroblasts (GFs) were seeded as a suspension at a concentration of approximately 0.5 × 10^6^ cells per Petri dish in sterile 60 mm Petri dishes. The cells were incubated at 37 °C in a 6% CO_2_ atmosphere until they reached confluence.

Once confluence was achieved, the culture medium was aspirated, and the scaffolds, along with the negative and positive controls, were placed at the center of each Petri dish. The negative control consisted of cells without a matrix, while the positive control was a piece of sterile gauze (1 × 1 cm) moistened with a 20% sodium dodecyl sulfate (SDS) solution. The samples and controls were subsequently covered with 4 mL of agar medium, composed of 1% agar in DMEM with 2% FBS, and incubated at 37 °C for 24 h.

After incubation, the cells were stained with 1% agar containing 0.2% neutral red. The samples were tested in triplicate, and the cytotoxicity was evaluated at 24 and 48 h based on cellular uptake of neutral red. This dye preferentially stains the acidic regions of viable cells, particularly lysosomes, resulting in red coloration. The cytotoxicity of the scaffolds was assessed by measuring the width of the unstained zone around the samples, which corresponds to areas where cell membranes were compromised. The response index was calculated as the ratio of the zone index to the lysis index.

#### 2.2.14. Contact Toxicity Testing (TCT)

Contact toxicity testing was conducted according to ISO 10993-5 [47]. A cell suspension of gingival fibroblasts (GFs) was prepared at a concentration of 0.5 × 10⁵ cells/mL. The polymer scaffolds, fabricated via 3D printing, were sterilized by UV radiation before testing. Each scaffold was placed at the center of a sterile 60 mm Petri dish and fixed in place with a sterile U-shaped glass. To each Petri dish, 1 mL of the cell suspension and 4 mL of DMEM were added.

Cell morphology was observed daily under an inverted microscope. At the end of the experiment, the cells were trypsinized using 0.25% trypsin and 0.02% EDTA, and the cell count was determined using a Bürker counting chamber. For evaluating cell proliferation in the presence of the tested polymer samples, negative and positive controls were included. The negative control (NC) consisted of a 1 × 1 cm sterile gauze piece, while the positive control (PC) was a sterile gauze piece moistened with a 20% sodium dodecyl sulfate (SDS) solution. Each sample was tested in triplicate.

## 3. Results and Discussion

The primary objective of this study was to develop a highly flexible material for 3D printing that is entirely bio-based and fully biodegradable under home composting conditions. To achieve elastic behavior, a two-phase system is typically required, as observed in thermoplastic elastomers (TPEs), where one phase provides rigidity and the other imparts softness. One viable approach involves combining an amorphous soft phase with a glass transition temperature (Tg) below the application temperature (e.g., below room temperature) with a rigid crystalline phase that has a melting temperature (Tm) above the application temperature (e.g., above room temperature).

However, highly crystalline polymers generally present challenges for 3D printing due to significant volumetric shrinkage during crystallization, which can cause pronounced warping. In the case of polyhydroxyalkanoates (PHAs), particularly polyhydroxybutyrate (PHB), controlling crystallinity through nucleating agents is difficult. This is because PHB-based PHAs crystallize rapidly and continue to do so even after processing, at ambient temperature. As a result, our approach focused on blending at least two polymers with distinctly different degrees of crystallinity. To maintain the fully bio-based and home-compostable nature of the final material, we excluded conventional petroleum-derived, non-biodegradable elastomers from consideration.

### 3.1. Rheology

**Figure 4:**
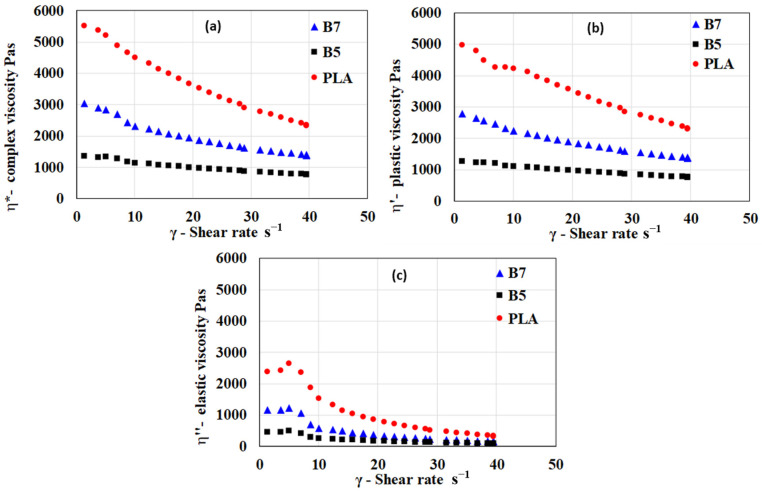
Typical flow curves of prepared blends expressed in terms of the dependency of the (**a**) complex viscosity, (**b**) plastic part of viscosity, and (**c**) elastic part of viscosity at 160 °C for PLA, B5, and B7 blends.

Rheological analysis of the polymeric blends was conducted to examine their viscoelastic properties in the molten state. Typical flow curves for a blend with the highest and a blend with the lowest viscosity are shown in Figure 4, together with a flow curve for PLA.

Prepared samples, including the PLA standard material, exhibit shear-thinning behavior. The melts predominantly display plastic behavior, with minimal elastic deformation during flow. In general, the viscosities of PHA blends are significantly lower than those of PLA, particularly at lower shear rates. Compared to PLA, PHA blends exhibit a lower contribution of elastic viscosity.

Unlike prior studies that primarily focus on the bulk rheological behavior of PHA-based blends [48,49], this research systematically investigates the correlation between crystallinity and viscosity reduction, demonstrating a significant improvement in low-shear-rate processability. The findings reveal that increasing the amorphous PHA content leads to a tunable shear-thinning profile, which can be tailored for specific 3D printing applications, ensuring better filament extrusion and layer adhesion.

**Figure 5:**
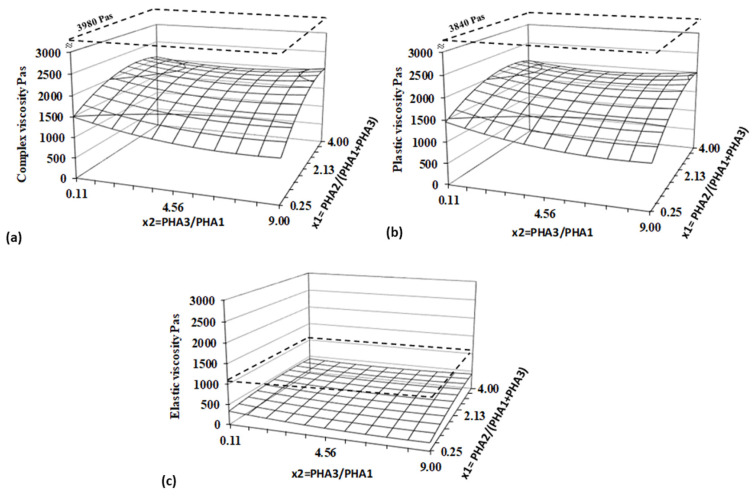
Response surfaces for (**a**) complex, (**b**) elastic, and (**c**) plastic viscosity evaluated for a shear rate of 15 s^−1^ and a temperature of 160 °C. Dashed planes represent values for PLA.

To assess the influence of composition on the rheological behavior of PHA blends, complex viscosity as well as the elastic and plastic components of viscosity at a shear rate of 15 s^−1^ were evaluated using the Design of Experiment (DoE) method. Regression analysis and statistical evaluation through ANOVA are presented in Table 3, and the response surfaces for all three viscosity components are depicted as 3D diagrams in Figure 5.

Statistical and regression analyses confirmed the high plasticity and low elasticity of the studied blends, with the response surface being significantly lower than the viscosity of PLA. Within the experimental range, the viscosity of PHA blends is strongly influenced by both factors, with the ratio of PHA_2_/(PHA_1_+PHA_3_) having the most significant effect. Amorphous PHA is the primary component determining the viscosity of the blend.

Compared to PLA, one of the most commonly used materials for 3D printing due to its suitable printability, the lower viscosity values observed in PHA blends suggest that these materials can be processed efficiently via 3D printing without significant challenges. In general, a low level of elastic flow in polymer melts results in reduced die swelling, which is beneficial for maintaining dimensional accuracy during extrusion-based processes [50,51]. Notably, the minimal elastic flow exhibited by PHA blends indicates a low likelihood of die swelling, further supporting their suitability for 3D printing applications.

**Figure 6:**
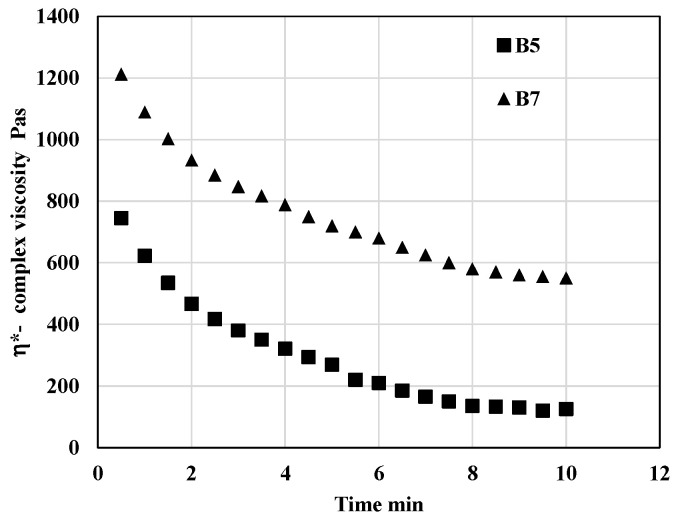
Dependency of complex viscosity on time during thermo-mechanical loading in oscillatory rheometer at 200 °C.

Rheological testing was also employed to evaluate the influence of blend composition on processing stability. The extent of degradation was quantified by monitoring the decrease in complex viscosity during the thermo-mechanical loading of the samples in an oscillatory rheometer. Experiments were conducted at 200 °C, which was identified as the optimal printing temperature for the majority of the prepared blends. Representative viscosity profiles obtained under the testing conditions described in Section 2.2.2 are presented in Figure 6. At the set testing conditions—primarily reflecting the selected printing temperature—the complex viscosity decreased over time due to thermal degradation. As shown in Figure 6, the blend composition had a pronounced effect on the viscosity behavior of the samples during processing.

**Figure 7:**
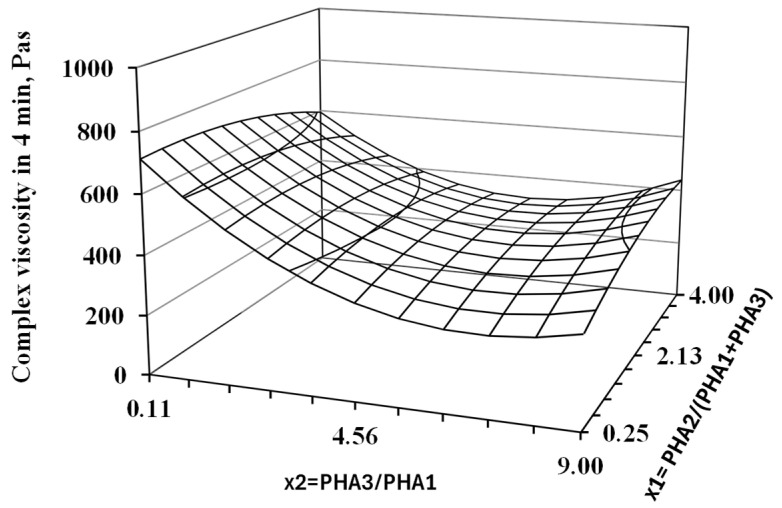
Response surfaces for complex viscosity after 4 min of thermo-mechanical loading of blend in oscillatory rheometer at 200 °C. The results of the regression and statistical analysis indicate that the ratio of the two crystalline PHAs (PHA3/PHA1) has a significant effect on degradation during processing at the printing temperature. Specifically, a higher proportion of PHA1 (corresponding to a lower value of the x_2_ factor) leads to a markedly more stable blend compared to those with a higher content of PHA3. In contrast, the concentration of the amorphous PHA2 appears to have a negligible influence on the processing stability of the studied PHA-based blends. Despite the noticeable effect of blend composition on degradation at 200 °C, all formulations were successfully processed using 3D printing. This is attributed to the short exposure time to elevated temperature during printing, typically on the order of seconds, which was insufficient to allow significant thermal degradation to occur.

Following the procedure outlined in Section 2.2.2, the complex viscosity at the fourth minute of testing was determined for all blend formulations. These values were subsequently analyzed using the Design of Experiment (DoE) methodology. The results of the regression analysis and statistical evaluation based on the DoE approach for all assessed parameters are summarized in Table 4. The corresponding response surface plot is presented in Figure 7.

### 3.2. Thermal Properties and Crystallinity

**Figure 8:**
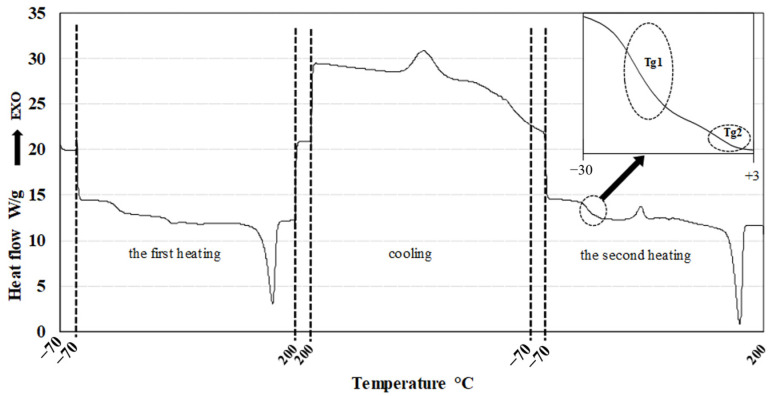
Typical DSC record of PHA blends.

**Figure 9:**
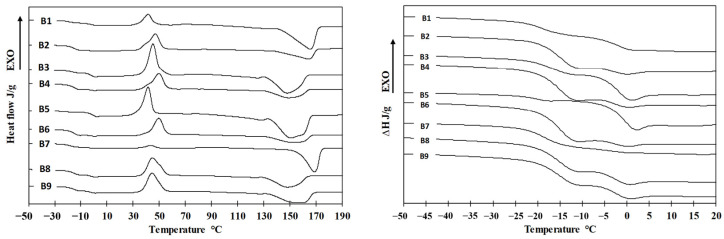
Overall DSC 2nd heating records (**left side**) and magnification for Tg region (**right side**) for blends B1–B9.

The thermal properties of the samples were assessed using differential scanning calorimetry (DSC). A representative DSC thermogram is shown in Figure 8. The first heating cycle was performed to erase the thermal history of the samples. Crystallization parameters were determined during the cooling phase, while the glass transition temperature (Tg), melting temperature (Tm), and melting enthalpy (ΔHm) were evaluated from the second heating cycle. DSC thermograms corresponding to the second heating of samples B1–B9 are presented in Figure 9.

Although each pure PHA exhibits a distinct Tg, the thermograms of the blends revealed only two Tg values, which differed from those of the individual components. This may indicate at least partial miscibility between PHA1 and the various PHA3 components. Alternatively, the observed results could be attributed to the limited resolution of the DSC method, which may not be sensitive enough to detect closely spaced Tg values (e.g., 5.1 °C and 2.4 °C). The measured Tg, Tm, and ΔHm values for all samples and pure components are summarized in Table 5.

Based on the measured thermal parameters—particularly the glass transition temperatures (Tg)—at least partial, and potentially full, miscibility between PHA3 and PHA1 in the blends can be inferred. This conclusion is supported by the significant shifts in Tg values observed for the blend components. For instance, while the Tg of PHA2 is −14.9 °C, blend B5 exhibits a Tg of −21.7 °C. None of the samples exhibited three distinct Tg values; rather, each blend displayed two Tg values, generally around −15 °C or lower. The presence of two Tg values indicates the existence of two distinct phases, each representing at least partial miscibility among two or more of the PHAs.

**Figure 10:**
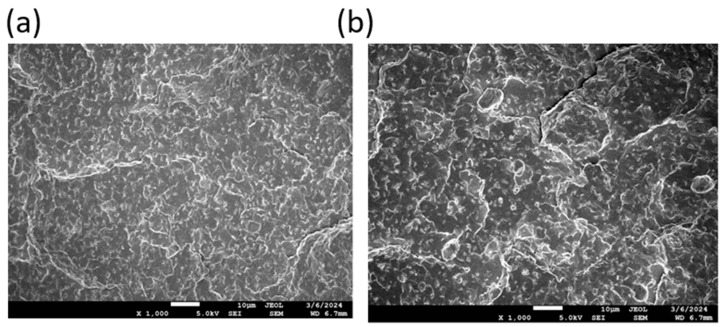
SEM photos of (**a**) for the samples B1)= and (**b**) for the sample **B2**, showing the fracture surface of filaments at 1000× magnification.

The existence of a two-phase system is further confirmed by scanning electron microscopy (SEM) analysis. Representative SEM images of selected filament samples are presented in Figure 10. These images reveal a two-phase morphology, characterized by a dispersion structure, confirming phase separation within the blends.

**Figure 11:**
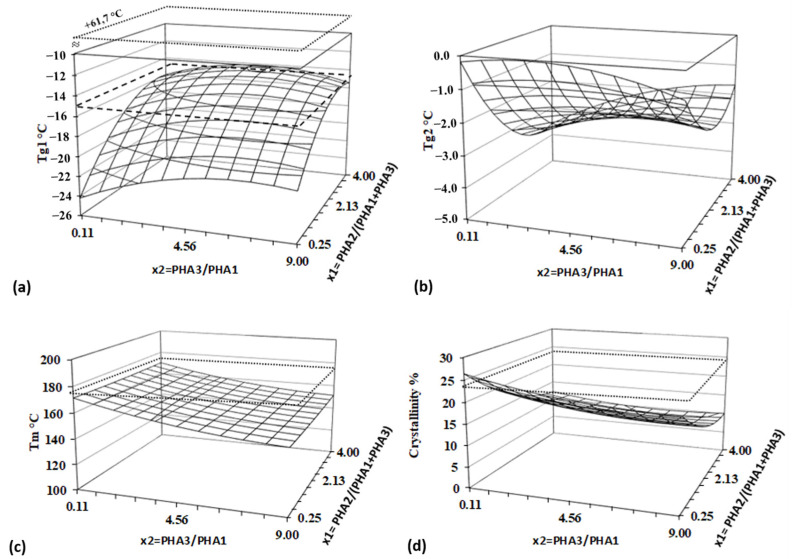
Response surfaces for thermal properties and crystallinity—(**a**) lower Tg 1, (**b**) higher Tg, (**c**) melting temperature, and (**d**) crystallinity. Dashed line represents value for PHA2, and dotted lines represent values for PLA. Regression and statistical analysis of the *Tg* values further confirm strong physical interactions between PHA polymers in the blend. A significant non-linear deviation from the original *Tg* of PHA2 (−14.9 °C) is observed for *Tg_1_*, which originally belongs to PHA2. Additionally, the two *Tg* values corresponding to PHA1 (+5.1 °C) and PHA3 (+2.4 °C) disappear from the DSC curves, replaced by a single *Tg_2_*, which does not exceed 0 °C.

All measured and calculated thermal parameters from Table 5 were analyzed using the Design of Experiment (DoE) method. The results, including regression analysis and ANOVA, are presented in Table 6, and response surfaces are illustrated in Figure 11.

*Tg_1_* represents the modified *Tg* of PHA2, and its shift to lower values indicates the existence of miscibility between PHA2 and/or PHA1 and PHA3 in the blends. This suggests that polymer segments interact with each other, behaving as plasticizers. The Tg_1_ temperature is strongly influenced by both independent variables, showing both linear and quadratic effects, which further supports molecular interactions among all three polymers. In contrast, *Tg_2_* depends only on factor x_1_, i.e., the content of amorphous PHA2, and is unaffected by the ratio of the two semicrystalline PHAs (PHA3/PHA1). This suggests that PHA1 and PHA3 form a single phase within the blend, which is partially miscible with PHA2. The strong dependence of Tg shifts on concentration ratios indicates partial miscibility of the blended polymers.

The melting temperature (*Tm*) is significantly influenced only by the ratio of semicrystalline polymers, decreasing as the PHA3 content increases, as PHA3 exhibits a lower Tm than PHA1. Figure 8 clearly shows that, despite the sufficiently different *Tm* values of PHA1 (166.7 °C) and PHA3 (148.6 °C), only a single melting peak is present, with no double peak formation. This suggests that PHA1 and PHA3 are miscible in the molten state, forming a mixed crystalline structure that is not influenced by the amorphous phase. Amorphous PHA2 primarily affects the crystallinity of the blend, with crystallinity decreasing as the PHA2 concentration increases. In contrast, the PHA3/PHA1 ratio has a much weaker impact on overall crystallinity.

### 3.3. Optimal Printing Temperature and Warping

**Figure 12:**
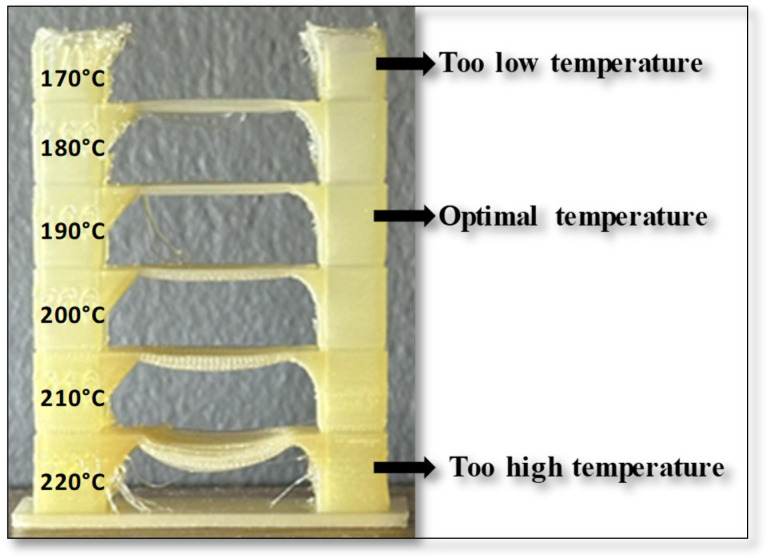
The 3D-printed temperature tower for sample B8 in temperature range from 170 °C to 220 °C.

Before 3D printing, the optimal printing temperature and warping properties, as crucial pre-printing features, were examined. It is important to note that these properties were not evaluated using the Design of Experiment (DoE) methodology. As described in the methodology section, the optimal 3D printing temperature was determined through a visual inspection of the printed temperature tower. An example of a 3D-printed temperature tower for the sample B8 is shown in Figure 12.

It is evident that the sample is not easily extrudable through the 3D printer nozzle at lower temperatures (170 °C), and the temperatures of 180 °C and 190 °C still do not yield optimal results. Moreover, at higher temperatures (210–220 °C), bending is observed at the center of the bridge. In contrast, the sample at 200 °C exhibits significantly improved 3D printing performance.

Consequently, the warping behavior during 3D printing was assessed for all samples within their optimal printing temperature range. The optimal printing temperatures, determined through temperature tower evaluation, along with the warping effects, are summarized in Table 7.

**Figure 13:**
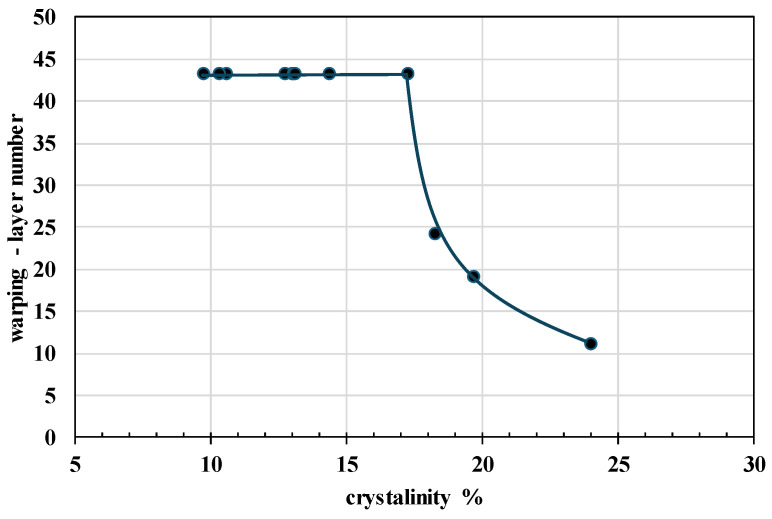
Dependency of warping effect expressed as layer number when detachment from bed appears on crystallinity. Value 43 for the warping layer number indicates no warping effect.

It is evident that the optimal printing temperature for all prepared PHA blends ranges between 200 °C and 210 °C, comparable to that of the standard (or even slightly lower). However, the warping tendency, expressed as the number of layers at which detachment from the print bed occurs, varies significantly across the blends. Lower values indicate poorer printability due to increased warping, and analysis revealed that warping behavior is strongly dependent on the crystallinity content of the material. Therefore, Figure 13 illustrates the evaluated relationship between warping and crystallinity.

**Figure 14:**
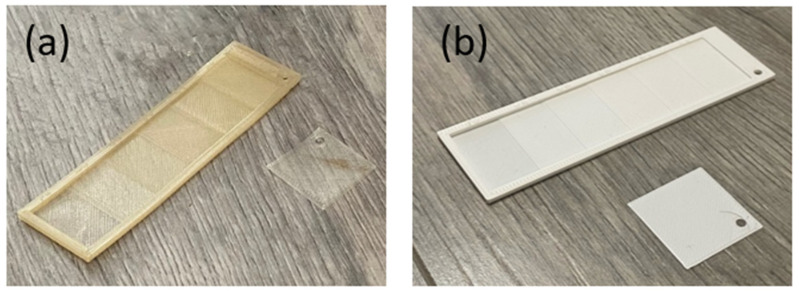
Typical structure of FDM 3D-printed (**a**) PHA-based blends (for sample B8) and (**b**) PLA sample in rectangular stair-shaped design.

The findings indicate that PHA-based blends with crystallinity levels below 18% exhibit no warping, which is essential for maintaining precision in biodegradable final product fabrication (Figure 14). By optimizing the composition of PHA blends, this study presents a material design strategy that improves both printability and mechanical performance. It is worth mentioning that, while previous research [36] has primarily assessed printability through qualitative methods, this study presents a material design strategy that improves both printability and mechanical performance.

In the subsequent step, rectangular stair-shaped specimens were printed according to the optimized conditions for each sample. Figure 14 illustrates a typical 3D-printed specimen, representing the architecture of a) all PHA-based samples and b) the PLA sample. Overall, all PHA-based blends were successfully 3D printed (compared to the slicer model (in Figure 3a,b)), demonstrating the excellent 3D printing capability of the novel PHA-based blends.

In fact, the optimal 3D printing temperature, low warping tendency, and precise 3D printing capability of these novel PHA-based blends position them as promising candidates for 3D printing processes, offering a competitive alternative to established materials.

### 3.4. Tensile Properties of Filaments and 3D-Printed Samples

**Figure 15:**
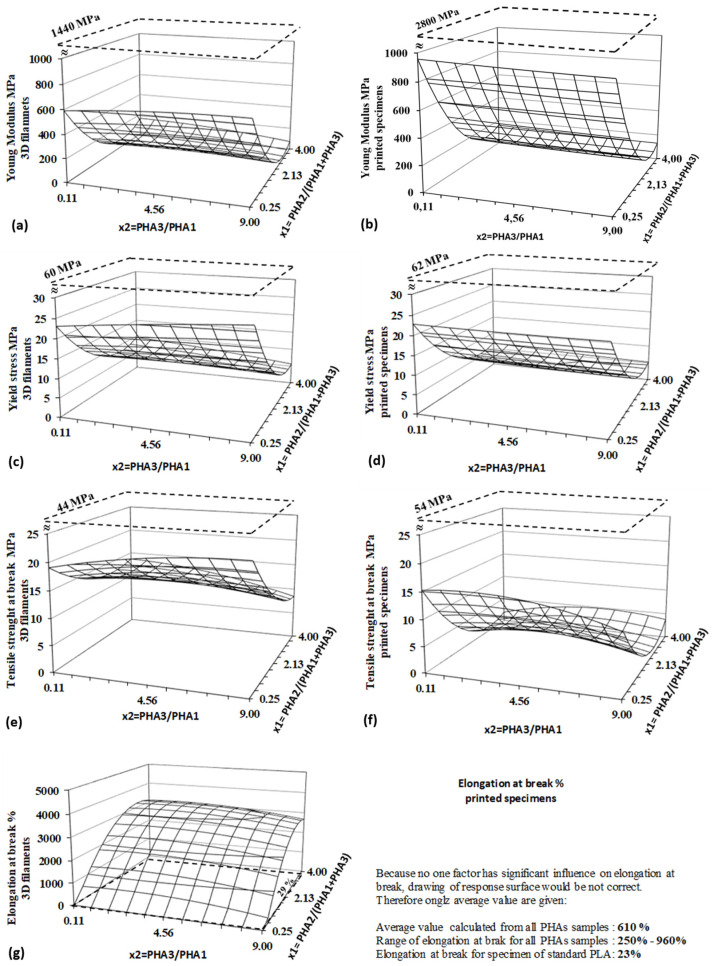
Response surfaces for tensile properties measured on 3D filaments (left side); (**a**) young modulus, (**c**) yield strength, (**e**) tensile strength at break, (**g**) elongation at break and on 3D-printed specimens (right side), (**b**) young modulus, (**d**) yield strength, (**f**) tensile strength at break. Dashed lines represent values for PLA.

The tensile properties of biodegradable blends were evaluated using two types of specimens: prepared filaments for 3D printing and printed dog-bone specimens for tensile testing. The properties measured on filaments represent the intrinsic material characteristics, whereas the values obtained from printed specimens also account for interlayer adhesion forces and the orientation of printed layers. Regression and statistical evaluations based on the DoE method for both specimen types are presented in Table 8, while the corresponding response surfaces are illustrated in Figure 15.

All tensile properties measured on 3D filaments are significantly influenced by factor x_2_, which corresponds to the content of amorphous PHA in the blend. These results demonstrate that the blends are highly flexible, with a high elongation at break. The elongation at break for PHA blends is dramatically higher compared to the negligible elongation observed for pure PLA filament. The elongation values for PHA blends can reach several thousand percent, in contrast to only twenty-nine percent for PLA. Given their high flexibility and significant elongation at break, these PHA blends exhibit promising mechanical properties that make them particularly suitable for soft tissue engineering applications, where materials need to closely mimic the flexibility and stretchability of biological tissues [54,55].

Logically, strength parameters—both yield stress and tensile strength at break—are significantly lower than those for pure standard PLA filament, but they remain sufficiently high for practical applications. The mechanical properties of 3D-printed specimens show slightly different results. While yield strength is solely dependent on the amorphous PHA content in the blend, the PHA3/PHA1 ratio does not have as significant an effect as in the filaments. The yield stress of printed specimens is comparable to that of the filaments, with layer adhesion and arrangement having no substantial influence on this property. In contrast, the tensile strength at break, as the ultimate property, is strongly influenced by the layer adhesion and layer arrangement. Final values for the tensile strength at break are significantly lower in the 3D-printed specimens compared to the filaments.

Regarding elongation at break, neither of the two factors significantly influences the results, due to high experimental variability, as reflected by the experimental standard deviation of ±348%. As a result, a response surface for elongation at break is not provided in Figure 15, as the surface lacks interpretive value. The high experimental error and the lower absolute values of both ultimate properties (tensile strength and elongation at break) for printed specimens, as compared to 3D filaments, can be attributed to layer delamination during the tensile tests at high deformation levels, which was observed during testing.

The results from the DoE evaluation of tensile testing demonstrate that the investigated PHA blends exhibit a wide range of mechanical properties, particularly a broad spectrum of flexibility, which is dependent on the blend composition. This offers a simple method for producing 3D printing materials with a diverse range of tensile properties, allowing for customization according to specific application requirements.

### 3.5. Hardness, Flexural Modulus, and Impact Strength

**Figure 16:**
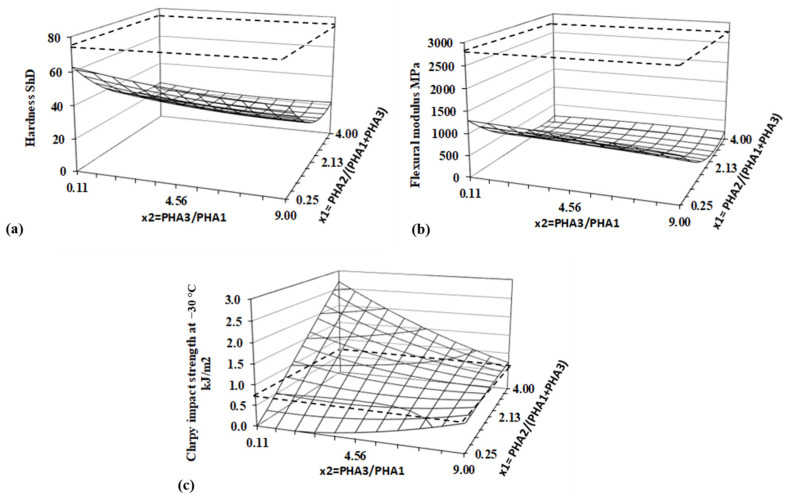
Response surfaces for (**a**) hardness, (**b**) flexural modulus, and (**c**) impact strength. Dashed lines represent values for PLA.

The flexibility of the material is well represented by hardness, while the toughness is reflected by the impact strength. The Shore D hardness, flexural modulus (measured in a three-point bending arrangement), and Charpy unnotched impact strength were measured following the procedures outlined in the Methods Section. The impact strength was tested at −30 °C, as at the ambient temperature of 23 °C, and all specimens were sufficiently flexible that, under the impact of the pendulum, they bent over the supporting holders to the opposite side without any defects. Regression and statistical evaluation values, based on the DoE method, for all evaluated parameters, are presented in Table 9, and the corresponding response surfaces are shown in Figure 16.

The hardness of PHA blends is significantly lower than that of PLA and is predominantly influenced by the content of amorphous PHA2, which causes a rapid decrease in hardness. The ratio of the two semicrystalline PHAs, PHA3/PHA1, has a weaker effect on the hardness of the blends. Logically, hardness slightly decreases as the relative concentration of the less crystalline PHA (PHA3) increases. While hardness primarily reflects the surface properties of the samples, the flexural modulus is more indicative of the bulk properties of the specimens. The flexural modulus of the PHA blends within the experimental range defined by the DoE is several times lower than that of PLA, with the lowest value being more than ten times lower. The flexural modulus of the studied PHA blends decreases rapidly with increasing amorphous PHA2 content, similar to hardness, but with a more pronounced curvature along the x_2_ axis.

An interesting behavior is observed in the impact strength, as determined by the unnotched Charpy impact test at −30 °C. While hardness and the flexural modulus are significantly influenced only by the amorphous content of the blend, the impact strength is determined by both factors. A relatively strong interaction between the two factors was confirmed through regression and statistical evaluation. The ratio of PHA3/PHA1 fine-tunes the crystallinity of the blend. It is evident that, when the blend contains a small amount of amorphous PHA2 (low values of x_2_), increasing the PHA3 content in the PHA3/PHA1 blend enhances the impact strength. Conversely, at higher PHA2 content, a higher PHA3/PHA1 ratio leads to a decrease in the impact strength. The highest impact strength in the investigated blends is observed at the highest PHA2 content and the highest PHA1 content within the PHA3/PHA1 ratio.

The results show that neither very high amorphous content nor very high crystallinity yields the highest impact strength. Instead, an optimal ratio of amorphous and crystalline content is necessary for the best performance. In any case, the investigated PHA blends offer several times higher impact resistance than standard PLA material.

Therefore, PHA blends can be regarded as a viable alternative to PLA, given their significant enhancements in hardness, flexural modulus, and impact strength.

### 3.6. Biodegradability in Real Home Compost Conditions

**Figure 17:**
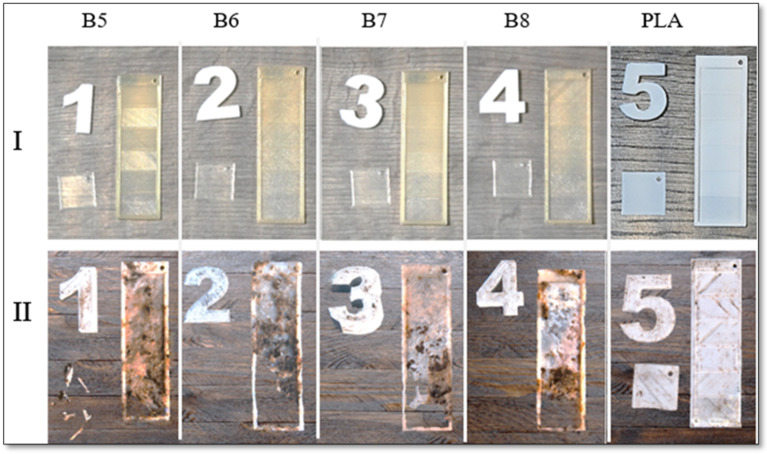
Visual inspection of composting of 3D-printed samples: (**I**) before composting and (**II**) after 2 months of home composting for samples of B5, B6, B7, B8, and PLA with crystallinity values of 24.1, 10.4, 18.3. 13.1, and 25%, respectively.

Biodegradability testing is essential for assessing the environmental impact of materials, particularly alongside the development of sustainable alternatives to conventional plastics. Following a two-month exposure to composting conditions, the samples were extracted from the compost. Photographs of all the tested samples, taken immediately upon removal, are presented in Figure 17.

It should be noted that, for home compostability in real conditions, samples from the DoE scheme were selected according to Table 10.

All small square-shaped samples with a thickness of 0.2 mm, printed from PHA blends, underwent complete decomposition during home composting and were no longer present in the compost. In contrast, rectangular stair-shaped specimens exhibited varied degradation behaviors. Notably, while all PHA-based samples demonstrated substantial degradation in the first step of the rectangular structures (0.2 mm thickness), the PLA sample remained intact, highlighting the difference in compostability between these materials.

Visual assessment of the exposed samples suggests that the higher proportion of amorphous PHA2 in sample B6, compared to its lower content in B5, contributed to a more rapid degradation rate. Crystallinity analysis further indicates that lower crystallinity enhances biodegradation, supporting the premise that the structural organization of the polymer influences its compostability. However, variations in the PHA3/PHA1 ratio in the other two samples demonstrate that a higher PHA1 content is more favorable for home composting. Despite sample B7 exhibiting greater crystallinity than sample B8, it degraded more rapidly, suggesting that biodegradation rates are influenced by factors beyond crystallinity alone.

**Figure 18:**
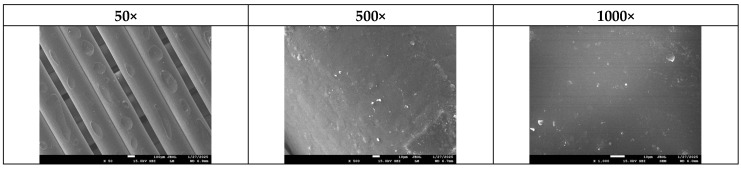
Typical SEM photos of surfaces of sample B5 before composting in different magnifications.

To gain a more detailed understanding of the degradation process, SEM analysis was conducted on all samples post-composting. Figure 18 presents the untouched surface of sample B5 before composting as a reference, with all other samples displaying a similar initial surface morphology.

**Figure 19:**
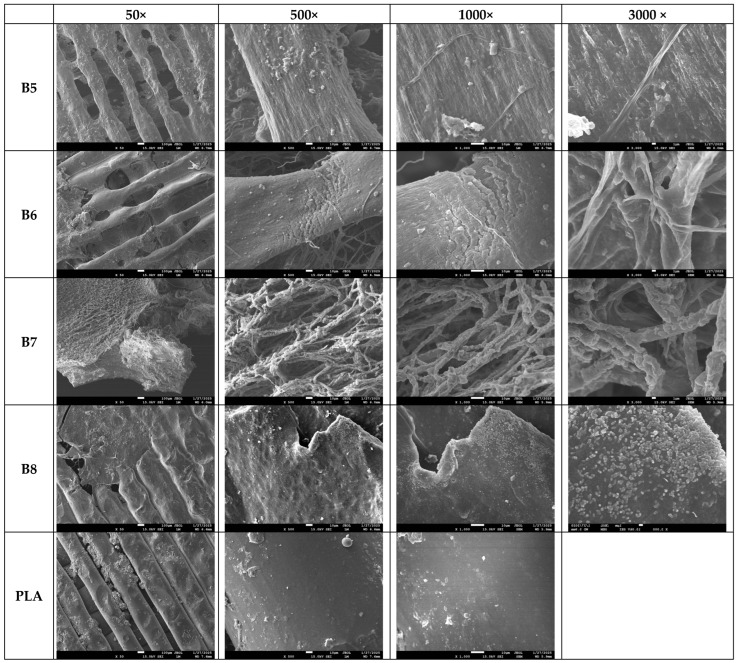
SEM photos of surfaces of samples B5–B8 and PLA after composting (the magnification is given in the first row).

The results depicted in Figure 19 present the degradation trends of the analyzed samples following a two-month home composting period. The SEM images captured at 50× and 500× magnifications reveal substantial decomposition in the PHA-based blends (samples B5–B8), as evidenced by significant material loss from the surface. In contrast, the PLA sample remains largely unaltered, exhibiting no visible signs of degradation under the same conditions. This difference in degradation behavior can be attributed to the intrinsic properties of polyhydroxyalkanoate (PHA) polymers, which are known for their rapid biodegradation in composting environments. PHA polymers are a class of biodegradable materials synthesized by various microorganisms as intracellular energy storage compounds. According to the literature, their microbial origin makes them highly susceptible to enzymatic degradation by a diverse range of bacteria and fungi present in composting systems [56,57].

**Figure 20:**
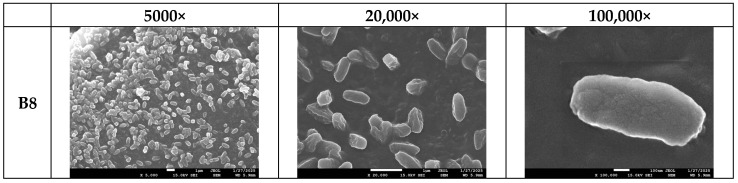
SEM photos for identification of bacteria colony on sample B8’s surface in different magnifications (the magnification is given above the photo).

Figure 19 shows that, at higher magnifications (1000× and 3000×), filamentous fungi are observed in small quantities on samples B5 and B6, while sample B7 shows a substantial portion of the surface covered by these fungi, indicating strong microbial adhesion to the sample surfaces. Furthermore, in Figure 20, sample B8, which exhibited substantial degradation, displays numerous bacterial colonies at 1000× to 3000× magnification. Higher magnifications (5000×, 20,000×, and 100,000×) were also examined to confirm the presence of these bacterial colonies.

The observed microbial activity aligns with existing literature emphasizing the pivotal role of bacteria and fungi in accelerating composting processes. Bacteria are the dominant microorganisms during all stages of composting, actively breaking down easily degradable organic materials. Their metabolic activities facilitate the decomposition of complex organic compounds, thereby enhancing the composting efficiency [58]. Furthermore, fungi, particularly filamentous types, are instrumental in degrading complexes that are more resistant to bacterial decomposition. Their extensive hyphal networks enable them to access and break down organic residues that are not readily available to bacteria, thus contributing significantly to the composting process [59]. The presence of bacterial colonies observed on sample B8 corroborates findings that these microorganisms are responsible for the rapid decomposition of materials during composting. Similarly, the extensive fungal coverage on sample B7 underscores the fungi’s role in breaking down complex organic substances, thereby facilitating the composting process. Therefore, the combination of microbial and fungal activity and the intrinsic biodegradability of PHA polymers facilitates the efficient breakdown of PHA-based materials and efficient decomposition compared to PLA in composting environments.

### 3.7. Cytotoxicity

The biodegradable and bio-based nature of the studied highly flexible polymer blends suggests their potential application in the fabrication of scaffolds for soft tissue engineering. Three-dimensional (3D) printing provides a promising approach for the precise customization of scaffold structures to accommodate patient-specific requirements. Blends B5 and B8 were selected for cytotoxicity evaluation as a fundamental assessment of their suitability for tissue engineering applications.

**Figure 21:**
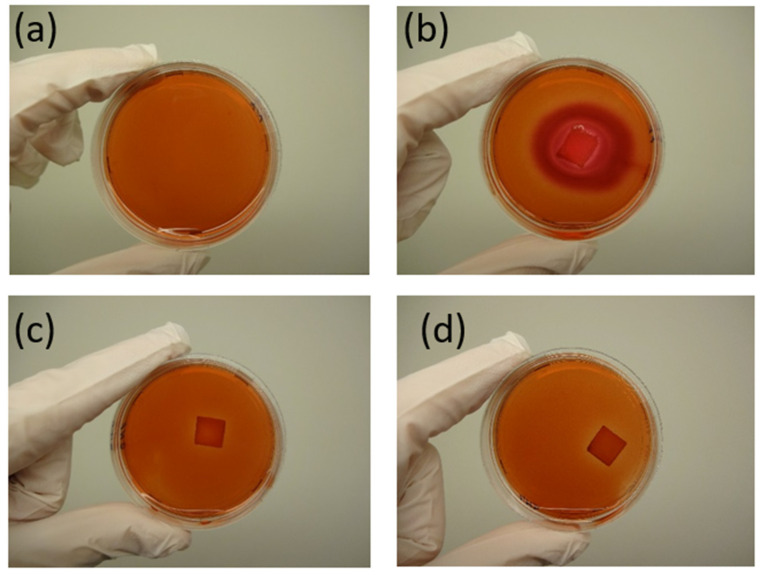
Results of the agar diffusion test for sample B5. (**a**) Negative control (only cells) (A (0/0), (**b**) positive control (gauze moistened with SDS) (B (4/4)), (**c**) sample B5 (C (0/0)), and (**d**) sample B8 (D (0/0)). The response index (zone/lysis) is indicated in parentheses.

Cytotoxicity was analyzed using the agar diffusion test, followed by an in vitro contact toxicity test, both performed in accordance with ISO 10993-5 [47], as detailed in Section 2.2. Figure 21 presents images of the negative and positive controls, as well as samples B5 and B8, after 24 h of exposure to the agar diffusion test.

**Figure 22:**
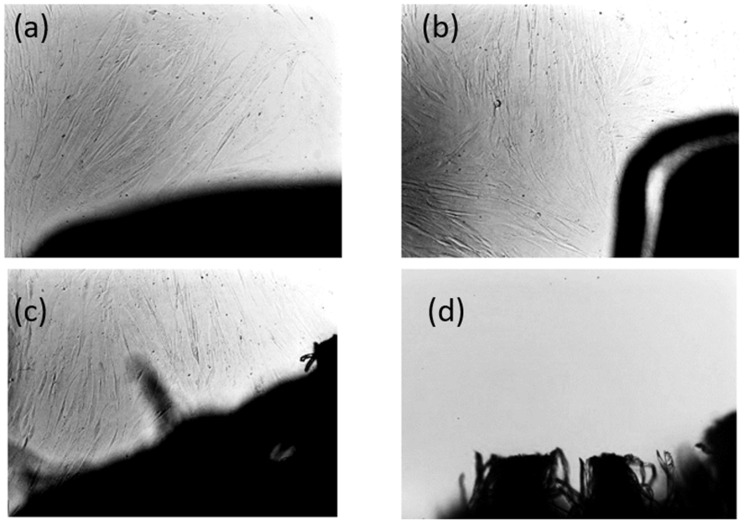
Microscopic images of cells cultivated in the presence of samples B5 (**a**) and B8 (**b**), alongside negative ((**c**)—gauze) and positive ((**d**)—gauze moistened with SDS) controls, after 72 h of the in vitro contact toxicity test (Magnification 10×).

**Figure 23:**
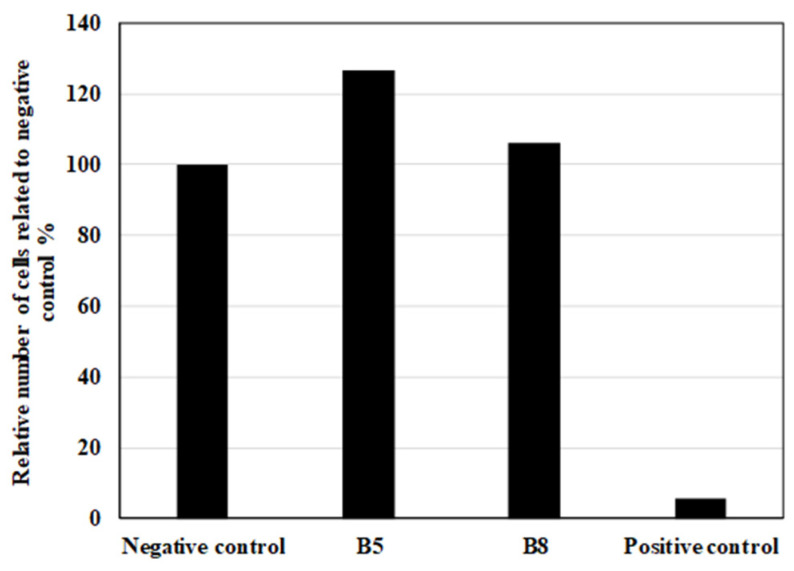
Cell proliferation after 72 h of in vitro contact toxicity test expressed in relative number (%) related to negative control with sterile gauze (100%).

The absence of noticeable discoloration in the periphery of samples B5 and B8 (Figure 21c) indicates a lack of cytotoxic response. The response index for zone/lysis was determined to be 0/0, confirming the non-cytotoxic nature of both polymer blends. This suggests that B5 and B8 do not adversely affect the viability of gingiva fibroblasts. Figure 22 and Figure 23 illustrate the results of the in vitro contact toxicity test, conducted over 72 h.

After 72 h, fibroblast cells exhibited normal growth and morphology in the vicinity of both B5 and B8 samples, comparable to the negative control (sterile gauze). No significant morphological alterations or signs of cytotoxicity were observed in fibroblasts exposed to the test materials. In contrast, in the positive control group, which contained SDS-treated gauze, extensive cell lysis was observed, with an almost complete absence of viable cells. The results are depicted in Figure 22.

To further assess the biocompatibility of the tested materials, quantitative cell proliferation analysis was conducted by determining the viable cell count after 72 h. As is depicted in Figure 23, the relative cell number was normalized to the negative control, allowing for a comparative assessment. The results indicate that samples B5 and B8 supported fibroblast proliferation at levels comparable to or exceeding those observed in the negative control group. This suggests that the presence of these materials may have a neutral or potentially supportive effect on cellular proliferation.

Based on these findings, the studied highly elastic, bio-based, biodegradable polymer blends exhibit non-cytotoxic behavior and can be considered promising candidates for the fabrication of 3D-printed scaffolds for tissue engineering applications. Further studies, including long-term biocompatibility assessments and in vivo evaluations, are warranted to validate their suitability for clinical applications.

## 4. Conclusions

This study introduces a new class of biodegradable polyhydroxyalkanoate (PHA) blends designed for Fused Deposition Modeling (FDM) 3D printing, offering an environmentally friendly alternative to conventional thermoplastics. By carefully adjusting the crystallinity of PHA components, we achieved better processability, reduced viscosity, and enhanced shear-thinning behavior, leading to smoother extrusion and improved layer adhesion. Compared to polylactic acid (PLA), these blends demonstrate greater flexibility, superior elongation at break, and enhanced impact resistance, addressing key limitations of existing biodegradable 3D printing materials. A major advantage of these formulations is their rapid degradation under real home composting conditions, where complete breakdown occurred within two months—far surpassing PLA, which remained intact. Additionally, cytotoxicity tests confirmed their biocompatibility, making them strong candidates for biomedical applications, particularly bioresorbable scaffolds for tissue engineering. This research provides one of the first in-depth analyses of how PHA crystallinity directly influences 3D printability, biodegradation, and mechanical performance, paving the way for more sustainable material innovations in fields such as packaging, agriculture, and medicine. Future studies should explore further refinement of these formulations and evaluate their long-term performance in demanding applications.

## Figures and Tables

**Table 1:** Conditions of experiment.

	Coded Levels						
	−1.141	−1	0	1	1.414		
Factor	Real values					Central value	step
x1	0.25	0.80	2.13	3.45	4	2.13	1.33
x2	0.11	1.412	4.56	7.70	9	4.56	3.14

**Table 2:** Composition of the blends in the DoE experiment.

Samples	Coded Values		Real Values	
	x1	x2	x1	x2
	PHA2/(PHA1+PHA3)	PHA3/PHA1	PHA2/(PHA1+PHA3)	PHA3/PHA1
B1	−1	−1	0.80	1.412
B2	+1	−1	3.45	1.412
B2	−1	+1	0.80	7.7
B4	+1	+1	3.45	7.7
B5	−1.414	0	0.25	4.56
B6	+1.414	0	4	4.56
B7	0	−1.414	2.13	0.11
B8	0	+1.414	2.13	9
B9	0	0	2.13	4.56
B10	0	0	2.13	4.56
B11	0	0	2.13	4.56
B12	0	0	2.13	4.56
B13	0	0	2.13	4.56

**Table 3:** Regression and statistical parameters evaluated for viscosity. (Values significant at 95% probability (*α*=0.05) are in bold.)

	Fcrit 0.05	Complex Viscosity	Plastic Viscosity	Elastic Viscosity
F1	6.9	88.5	91.2	52.8
F2	6.6	23.0	23.4	18.5
FLF	6.6	1.0	1.0	0.9
	sE+/−	62	59	17
	sLF+/−	61	60	16
	b0	1817	1781	361
	b1	278	272	55
	b2	−81	−77	−25
	b11	−160	−156	−34
	b12	54	51	18
	b22	81	77	25

**Table 4:** Regression and statistical parameters evaluated for complex viscosity in 4th minute. (Values significant at 95% probability (*α* = 0.05) are in bold).

	Fcrit 0.05	Complex Viscosity in 4th min
F1	6.9	**121.4**
F2	6.6	**76.7**
FLF	6.6	**22.7**
	sE+/−	18.2
	sLF+/−	22.7
	b0	366.8
	b1	2.9
	b2	**−100.4**
	b11	**−21.8**
	b12	**32.3**
	b22	**95.9**

**Table 5:** Tg, Tm, ΔHm, and crystallinity values of prepared blends and their components.

Sample	Tg 1 (°C)	Tg 2 (°C)	Tm (°C)	ΔHm (J/g)	^1^ Crystallinity (%)
PHA1	5.1	-	166.7	75.9	52.0
PHA2	−14.9	-	-	-	-
PHA3	2.4	-	148.6	52.8	36.2
B1	−19.7	−1.1	166.3	28.9	19.8
B2	−14.2	−2.7	165.4	15.5	10.6
B3	−18.6	−1.7	151.2	25.3	17.3
B4	−14.5	−2.1	151.2	14.3	9.8
B5	−21.7	0.3	156.9	35.1	24.07
B6	−14.6	−2.6	157.3	15.1	10.4
B7	−18.9	−3.4	170.4	26.7	18.3
B8	−15.7	−2.1	149.9	19.1	13.1
B9	−14.5	−1.3	157.8	19.1	13.1
B10	−15.2	−2.4	153.3	18.7	12.8
B11	−15.6	−2.6	163.0	21.1	14.4
B12	−15.2	−2.6	155.7	19.2	13.1
B13	−15.6	−2.8	153.2	19.2	13.1
PLA ref.	61.16	-	151.8	23.4	25.0

^1^ The crystallinity was evaluated based on the enthalpy of melting ΔHm, and the ΔH0 enthalpy for melting a fully crystalline polymer was assumed to be 146 J/g for PHA and 93.6 J/g for PLA, as referenced in studies [52,53].

**Table 6:** Regression and statistical parameters evaluated for thermal properties and crystallinity. Values significant at 95% probability (α = 0.05) are in bold.

	Fcrit 0.05	Tg1	Tg2	Tm	Crystallinity
F1	6.9	154.72	154.72	12.36	212.69
F2	6.6	26.51	26.51	0.48	13.35
FLF	6.6	7.17	7.17	0.01	9.43
	sE+/−	0.41	0.41	4.1	0.64
	sLF+/−	1.10	1.10	0.4	1.98
	b0	−15.24	−15.24	156.6	13.31
	b1	2.46	2.46	0.0	−4.50
	b2	0.68	0.68	−7.1	−1.33
	b11	−1.20	−1.20	0.3	1.43
	b12	−0.34	−0.34	0.3	0.41
	b22	−0.79	−0.79	1.8	0.67

**Table 7:** Values of optimal printing temperature as well as values of warping effect.

	Warping	Optimal Printing Temperature
Sample	(Layer Number)	(°C)
B1	19	200
B2	43	200
B3	43	200
B4	43	205
B5	11	200
B6	43	210
B7	24	200
B8	43	200
B9	43	200
B10	43	200
B11	43	205
B12	43	200
B13	43	205
PLA	43	210

**Table 8:** Regression and statistical parameters evaluated for tensile properties of 3D filaments and 3D-printed specimens. Values significant at 95% probability (α = 0.05) are in bold.

		3D Filaments			3D-Printed Specimens		
	Fcrit 0.05	*σ_Y_* ^1^	*σ_B_* ^2^	*ε_B_* ^3^	*σ* *_Y_*	*σ* *_B_*	*ε_B_*
F1	6.9	**260.6**	**26.7**	**30.9**	**230.6**	**170.8**	2.9
F2	6.6	**49.5**	4.6	5.1	**42.6**	**76.2**	0.6
FLF	6.6	**7.4**	**8.5**	0.4	2.1	**95.9**	0.5
	sE+/−	0.77	1.4	420	0.79	0.58	348
	sLF+/−	2.09	4.0	249	1.15	5.69	240
	b0	**9.89**	**14.0**	**2662**	**7.64**	**4.14**	**610**
	b1	**−6.20**	**−3.5**	**1158**	**−5.96**	**−3.78**	289
	b2	−0.02	−0.2	−142	−0.30	−0.35	−60
	b11	**3.42**	**1.6**	**−623**	**3.35**	**2.99**	−90
	b12	−1.00	−1.1	−8	−0.17	0.72	−44
	b22	−0.14	−0.5	−94	0.09	**−0.96**	136

^1^ *σ_Y_*—yield stress, ^2^ *σ_B_*—tensile strength at break, ^3^ *ε_B_*—elongation at break.

**Table 9:** Regression and statistical parameters evaluated for hardness, flexural modulus, and impact strength according to the Charpy unnotched method at −30 °C. (Values significant at 95% probability (α=0.5) are in bold.)

	Fcrit 0.05	Hardness	Flexural Modulus	Impact Strength at −30 °C
F1	6.9	210.2	367.0	43.1
F2	6.6	29.3	79.6	6.3
FLF	6.6	3.4	11.8	3.3
	sE+/−	1.7	37	0.17
	sLF+/−	3.1	126	0.30
	b0	26.6	290	0.89
	b1	−11.8	−351	0.50
	b2	−41	−0.22	
	b11	5.9	210	−0.02
	b12	1.0	36	−0.34

**Table 10:** Selected samples for home compostability testing.

Sample Number in Compost	Sample Number in DoE	Sample Description
1	B5	Sample with the lowest PHA2 amorphous PHA content at medium PHA3/PHA1 ratio
2	B6	Sample with the highest PHA2 amorphous PHA content at medium PHA3/PHA1 ratio
3	B7	Sample with lowest PHA3/PHA1 ratio at medium PHA2 content
4	B8	Sample with highest PHA3/PHA1 ratio at medium PHA2 content
5	PLA	Commercial PLA sample

## Data Availability

The original contributions presented in this study are included in the article. Further inquiries can be directed to the corresponding author.
